# Enhancing fraction measured using dynamic contrast-enhanced MRI predicts disease-free survival in patients with carcinoma of the cervix

**DOI:** 10.1038/sj.bjc.6605415

**Published:** 2009-11-17

**Authors:** S B Donaldson, D L Buckley, J P O'Connor, S E Davidson, B M Carrington, A P Jones, C M L West

**Affiliations:** 1 North Western Medical Physics, The Christie, Wilmslow Road, Withington, Manchester M20 4BX, UK; 2 Imaging Science and Biomedical Engineering, University of Manchester, Oxford Road, Manchester M13 9PT, UK; 3 Division of Medical Physics, University of Leeds, Worsley Building, Clarendon Way, Leeds LS2 9JT, UK; 4 Department of Radiology, The Christie, Wilmslow Road, Withington, Manchester M20 4BX, UK; 5 Department of Clinical Oncology, The Christie, Wilmslow Road, Withington, Manchester M20 4BX, UK; 6 School of Cancer Imaging Sciences, University of Manchester, The Christie, Withington, Manchester M20 4BX, UK

**Keywords:** enhancing fraction, dynamic contrast-enhanced MRI, cervix cancer, radiotherapy, imaging biomarkers

## Abstract

**Background:**

There is a need for simple imaging parameters capable of predicting therapeutic outcome.

**Methods:**

This retrospective study analysed 50 patients with locally advanced carcinoma of the cervix who underwent dynamic contrast-enhanced MRI before receiving potentially curative radiotherapy. The proportion of enhancing pixels (*E*_F_) in the whole-tumour volume post-contrast agent injection was calculated and assessed in relation to disease-free survival (DFS).

**Results:**

Tumours with high *E*_F_ had a significantly poorer probability of DFS than those with low *E*_F_ (*P*=0.011).

**Interpretation:**

*E*_F_ is a simple imaging biomarker that should be studied further in a multi-centre setting.

Carcinoma of the cervix is the second most common cancer in women and a significant cause of mortality worldwide (Monk *et al*, 2009). Outcome of patients with locally advanced disease has improved in recent years with the adoption of concurrent chemoradiotherapy (Green *et al*, 2001). However, the overall 5-year survival rate is still only around 60% and the use of concurrent chemotherapy may increase the risk of late toxicity (Spensley *et al*, 2009). Consequently, there is a need to predict, before treatment, those patients likely to respond to radiotherapy alone and those who might benefit from combination therapy and/or the addition of novel targeted therapeutic agents.

There is interest in developing imaging biomarkers that predict cancer treatment outcome. Dynamic contrast-enhanced magnetic resonance imaging (DCE-MRI) can be used to estimate microvascular parameters such as tissue perfusion and capillary permeability. Some of the parameters have been shown to correlate with histological measurements of angiogenesis (Mayr *et al*, 1996; Hawighorst *et al*, 1999) and tumour oxygenation (Cooper *et al*, 2000) and have had some success in predicting treatment outcome (Loncaster *et al*, 2002). Although tracer kinetic analysis of DCE-MRI data results in the estimation of physiologically relevant parameters, the requirement for high-temporal resolution data – enabling an arterial input function (Parker *et al*, 2006) to be obtained – and pre-contrast *T*_1_ measurements make DCE-MRI studies difficult to implement. There is interest, therefore, in developing simple imaging biomarkers of tumour physiology.

Enhancing fraction (*E*_F_) assessed using DCE-MRI or CT has been presented as a simple method of assessing whole-tumour vascularity. Enhancing fraction is the proportion of tissue within a tumour that enhances and has been shown to predict treatment outcome in ovarian (O’Connor *et al*, 2007) and other solid tumours (Mullamitha *et al*, 2007) and has been used to assess treatment efficacy of an anti-angiogenic agent (Jayson *et al*, 2005). The aim of this study was to investigate whether *E*_F_ measured with DCE-MRI was able to predict treatment outcome in patients with carcinoma of the cervix.

## Materials and methods

### Patients

Approval from a local ethics committee was obtained and all patients gave informed consent. Fifty patients with locally advanced carcinoma of the cervix underwent pre-treatment MRI scans. Patients subsequently received 40–45 Gy external beam radiotherapy (EBRT) to the pelvis – 20 fractions over 28 days – followed by low dose rate intracavitary brachytherapy (22.5−32.5 Gy). Disease-free survival (DFS) was calculated from the date of start of radiotherapy to date of disease recurrence or latest follow-up date where no recurrence occurred.

### MR protocol

MRI scans were performed on a 1 T Siemens Magnetom Impact (Siemens Healthcare, Frimley, UK). Staging scans were carried out as previously described (Cooper *et al*, 2000; Loncaster *et al*, 2002). Dynamic scans used a sagittal 2D *T*_1_-weighted FLASH sequence (TR/TE=130/6.5 ms, FOV=290 × 290 × 5 mm, matrix=256 × 256, NSA=1, *α*=70°) with a temporal resolution of 25 s. One sequence of nine contiguous slices covering the whole-tumour volume was obtained before and seven after injection of 0.1 mmol kg^−1^ body weight Gd-DTPA.

### Data analysis

Regions of interest (ROIs) encompassing the whole tumour were defined by an experienced radiologist on the pre-contrast *T*_2_-weighted images. Regions of interest were transferred to the dynamic data series. Data analysis was carried out using IDL (Research Systems Inc., Boulder, CO, USA). Tumour volumes were calculated by multiplying the number of pixels in the ROI by the voxel dimensions. A threshold for enhancing pixels was defined as a signal change greater than three times the standard deviation (s.d.) of signal values over the whole-tumour volume calculated in the pre-contrast image. The number of pixels that exceeded this threshold at 25 and 50 s post-contrast agent injection was calculated and divided by the total number of pixels in the tumour ROI to obtain *E*_F_ at 25 and 50 s, respectively.

Pearson correlations between tumour volume and *E*_F_ at 25 and 50 s were investigated using SPSS (SPSS Inc., Chicago, IL, USA). Spearman correlations between tumour stage and tumour volume, *E*_F_ at 25 and 50 s were assessed. Tumours were stratified into two groups – those with tumour volume, stage and *E*_F_ above or below the median. Kaplan–Meier curves were produced and a log-rank test performed to investigate significant differences in DFS. A significance level of 0.05 with a Bonferroni correction for multiple comparisons was used. A Fisher's exact test was used to assess differences in *E*_F_ at 25 s in tumours of different size, stage, nodal status and age of the patient.

## Results

Patient details are shown in Table 1. The median age of the 50 women was 63 years (range 29–82 years). The median tumour volume was 49.9 cm^3^ (range 6.8–184.2 cm^3^); 46 tumours were squamous cell carcinomas, 3 were adenosquamous cell carcinomas and 1 was adenocarcinoma; 26 and 24 patients had negative and positive nodal status, respectively. The median follow-up time in surviving patients was 105 months (range 86–128 months). A total of 32 patients had recurrence of their disease at the time of writing – 8 patients had local recurrence only, 9 patients had distant recurrence only, 13 had local and distant recurrence and 2 had residual disease after treatment.

**Figure 1:**
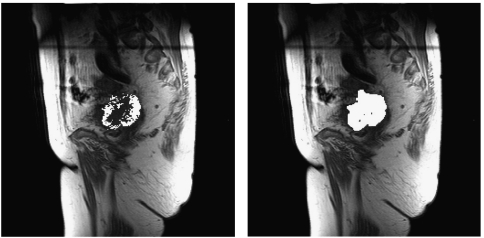
*E*_F_ in a cervix tumour (squamous cell carcinoma, stage IIIB) at 25 (36.5%) and 50 s (95.9%), respectively. The pre-treatment *E*_F_ at 25 s was greater than the median and the patient subsequently developed local recurrence and metastases and died from their disease 17 months after diagnosis.

Figure 1 shows the distribution of enhancing pixels for *E*_F_ calculated at 25 and 50 s, respectively, for one patient. Enhancing fraction at 25 s post-contrast was higher in larger tumours (*P*<0.01). The results of correlations are shown in Table 2. There was a weak positive correlation between tumour volume and *E*_F_ at 25 s (*r*=0.37, *P*<0.01). There were no associations between *E*_F_ at 50 s post-contrast and any clinicopathological parameter.

**Figure 2:**
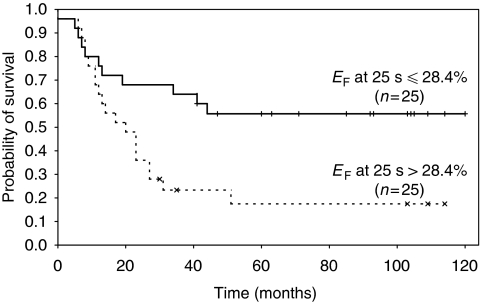
Kaplan–Meier curves showing the relationship between disease-free survival (DFS) and *E*_F_ measured at 25 s post-contrast agent injection using an enhancement threshold of three times the s.d. in the baseline signal measured over the whole-tumour volume. Patients were stratified by the median *E*_F_ at 25 s that was 28.4%. Numbers of patients in each arm and statistical significance are indicated.

Figure 2 shows the Kaplan–Meier curves for patients stratified by median *E*_F_ at 25 s (28.4%). There was a significant difference between DFS in patients with high and low tumour *E*_F_ at 25 s (*P*=0.011). Enhancing fraction calculated at 50 s was not associated with DFS. In this cohort of patients neither baseline tumour volume (*P*=0.88) nor disease stage (*P*=0.09) had prognostic significance.

## Discussion

A total of 50 patients with carcinoma of the cervix underwent DCE-MRI scans before receiving EBRT. We calculated *E*_F_ at two time points to examine the prognostic significance of *E*_F_ in this patient group. Patients with tumours with an *E*_F_ at 25 s greater than the median had a significantly poorer DFS than those with low *E*_F_, concurring with a similar study in ovarian cancer (O’Connor *et al*, 2007). This finding is consistent with high *E*_F_ reflecting more vascular and angiogenic tumours that are more likely to recur than those with low *E*_F_. The observation is consistent with work (Hawighorst *et al*, 1998) in which high *k*_ep_ – a rate constant describing the transfer of contrast agent between the vascular and the interstitial space – was indicative of a poor prognosis.

Other studies in cervical cancer have shown that high amplitude of enhancement is related to improved patient survival. Previous work involving a subset of this patient group (Loncaster *et al*, 2002) showed that high *A*^H^ – a parameter dependent on the size of the interstitial space (Hoffman *et al*, 1995) – was associated with improved patient survival. Similarly, Mayr *et al* (1996) showed that high relative signal intensity (rSI) – the ratio of post- to pre-contrast signal – resulted in improved prognosis. These results suggest that *E*_F_ measured early in the dynamic time series provides different physiological information from *A*^H^ and rSI. We postulate that *E*_F_ at 25 s is likely to relate to tissue perfusion whereas *A*^H^ and rSI are associated with the size of the interstitial space. A low *E*_F_ may also be consistent with a large hypoxic fraction; however, further work is needed to characterise the precise relationship, if any, between *E*_F_ and hypoxic fraction. Recent work (Zahra *et al*, 2009) showed a weak correlation between *k*_ep_ and tumour regression measured at 5 weeks after radiotherapy, with high enhancement being associated with better tumour regression; however, the results of long-term follow-up were not presented.

This study involved the retrospective analysis of data collected between July 1997 and August 2000. Few dynamic data points were acquired and the 25-s temporal resolution precluded accurate tracer kinetic analysis. The MRI signal threshold above which pixels are said to have enhanced was calculated using the s.d. of the baseline signal in the whole-tumour ROI due to the lack of multiple baseline (pre-contrast) data points. Despite this limitation, we showed a significant relationship between *E*_F_ and patient outcome. This finding agrees with other studies (Mullamitha *et al*, 2007; O’Connor *et al*, 2007) where the precise method of defining enhancement varied, showing that only a few dynamic data points and an accurate assessment of baseline tumour volume are required for calculation of *E*_F_. Although *E*_F_ is simple to calculate when compared with tracer kinetic analysis, precise definitions of enhancement will vary with the MR protocol being used, magnet strength, contrast agent dose and rate of administration. These factors may affect the precision of the measurement but *E*_F_ at 25 s should still reflect tissue perfusion, as hypothesised, and be capable of predicting DFS. Studies with more baseline signal data have used statistical techniques (O'Connor *et al*, 2009) to define enhancing pixels. Further work in larger prospective studies is therefore required to establish how best to define a threshold; however, our results add weight to the development of *E*_F_ as an imaging biomarker applicable to routine clinical use.

The patients included in this study were treated up to 10 years ago, when radiotherapy alone was commonly prescribed as treatment of choice. Current best practice recommends concurrent chemoradiotherapy for patients with carcinoma of the cervix, so it is important to test whether *E*_F_ has the same prognostic potential in this patient group. However, patients with locally advanced disease still receive radiotherapy alone if they have inadequate renal function for chemotherapy.

In conclusion, the results of this retrospective study suggest that *E*_F_ measured using DCE-MRI is capable of predicting DFS in patients with carcinoma of the cervix. High *E*_F_ was associated with poor prognosis. Enhancing fraction is a simple imaging biomarker and does not rely on sophisticated MRI techniques or data analysis. Confirmation in further datasets should be carried out to validate *E*_F_ as a biomarker of prognosis in locally advanced carcinoma of the cervix.

## Figures and Tables

**Table 1:** Summary of the distribution of patients as stratified by median *E*_F_ at 25 s (28.4%)

**Parameter**	***n***	**Low *E*_F_ at 25 s (*n*)**	**High *E*_F_ at 25 s (*n*)**	***P*-value^a^**
*Stage*				0.047
I	1	1	0	
II	25	13	12	
III	23	10	13	
IVA	1	1	0	
				
*Tumour volume*				<0.01^b^
Less than median	25	17	8	
Greater than median	25	8	17	
				
*Age (years)*				0.157
⩽62	25	11	14	
>62	25	14	11	
				
*Nodal status*				0.018
Negative	26	17	9	
Positive	24	8	16	

a Assessed by Fisher's exact test.

b Significant at the 0.05 level with Bonferonni correction applied for multiple comparisons.

**Table 2:** Results of correlations between parameters

	***E*_F_ at 25 s**	***E*_F_ at 50 s**	**Tumour volume**	**Tumour stage**
*E*_F_ at 25 s	—	0.596^a^	0.374^a^	−0.025
*E*_F_ at 50 s	0.596^a^	—	0.296	−0.011
Tumour volume	0.374^a^	0.296	—	0.148
Tumour stage	−0.025	−0.011	0.148	—

Pearson correlations are presented in all cases except for correlations between parameters with tumour stage that used a Spearman's test.

a Significant at 0.05 level with Bonferroni correction applied for multiple comparisons.
